# Oral Administration of Oxytocin, Like Intranasal Administration, Decreases Top-Down Social Attention

**DOI:** 10.1093/ijnp/pyac059

**Published:** 2022-09-02

**Authors:** Qian Zhuang, Xiaoxiao Zheng, Shuxia Yao, Weihua Zhao, Benjamin Becker, Xiaolei Xu, Keith M Kendrick

**Affiliations:** The Clinical Hospital of Chengdu Brain Science Institute, MOE Key Laboratory for Neuroinformation, Center for Information in Medicine, University of Electronic Science and Technology of China, Chengdu, China; Center for Cognition and Brain Disorders, The Affiliated Hospital of Hangzhou Normal University, Hangzhou, Zhejiang Province, China; Brain Cognition and Brain Disease Institute, Shenzhen Institute of Advanced Technology, Chinese Academy of Sciences, Shenzhen, China; The Clinical Hospital of Chengdu Brain Science Institute, MOE Key Laboratory for Neuroinformation, Center for Information in Medicine, University of Electronic Science and Technology of China, Chengdu, China; The Clinical Hospital of Chengdu Brain Science Institute, MOE Key Laboratory for Neuroinformation, Center for Information in Medicine, University of Electronic Science and Technology of China, Chengdu, China; The Clinical Hospital of Chengdu Brain Science Institute, MOE Key Laboratory for Neuroinformation, Center for Information in Medicine, University of Electronic Science and Technology of China, Chengdu, China; The Clinical Hospital of Chengdu Brain Science Institute, MOE Key Laboratory for Neuroinformation, Center for Information in Medicine, University of Electronic Science and Technology of China, Chengdu, China; The Clinical Hospital of Chengdu Brain Science Institute, MOE Key Laboratory for Neuroinformation, Center for Information in Medicine, University of Electronic Science and Technology of China, Chengdu, China; School of Psychology, Shandong Normal University, Jinan, Shandong Province, China; The Clinical Hospital of Chengdu Brain Science Institute, MOE Key Laboratory for Neuroinformation, Center for Information in Medicine, University of Electronic Science and Technology of China, Chengdu, China

**Keywords:** Intranasal oxytocin, oral oxytocin, antisaccade task, face emotion, social attention

## Abstract

**Background:**

The neuropeptide oxytocin (OXT) modulates social cognition by increasing attention to social cues and may have therapeutic potential for impaired social attention in conditions such as autism spectrum disorder. Intranasal administration of OXT is widely used to examine the drug’s functional effects in both adults and children and is assumed to enter the brain directly via this route. However, OXT can also influence brain function through increased blood concentrations, and we have recently shown that orally (lingual) administered OXT also modulates neural responses to emotional faces and may be better tolerated for therapeutic use. Here, we examine whether 24 IU OXT administered orally can facilitate social attention.

**Methods:**

In a randomized, placebo-controlled pharmacologic study, we used a validated emotional antisaccade eye-tracking paradigm to explore the effects of oral OXT on bottom-up and top-down attention processing in 80 healthy male participants.

**Results:**

Our findings showed that in terms of top-down attention, oral OXT increased errors for both social (angry, fearful, happy, sad, and neutral emotion faces) and nonsocial stimuli (oval shapes) in the antisaccade condition but increased response latencies only in the social condition. It also significantly reduced post-task state anxiety, but this reduction was not correlated with task performance. A comparison with our previous intranasal OXT study using the same task revealed that both routes have a similar effect on increasing antisaccade errors and response latencies and on reducing state anxiety.

**Conclusions:**

Overall, our findings suggest that oral administration of OXT produces similar effects on top-down social attention control and anxiety to intranasal administration and may therefore have therapeutic utility.

**Significance Statement**

Intranasally administered oxytocin (OXT) can enhance social attention, which may be beneficial for the treatment of autism spectrum disorder. It is thought to do so primarily through direct entry into the brain. However, oral (lingual) administration of OXT, which can influence brain function only through increased peripheral concentrations, modulates neural responses to emotional faces and is potentially better tolerated for therapeutic use. The current study used a validated emotional antisaccade eye-tracking paradigm to demonstrate that orally administered OXT (24 IU) also influences top-down attention control in terms of increased errors for social and nonsocial stimuli, increased response latencies only for social stimuli in the antisaccade condition, and reduced post-task state anxiety. A comparison with our previous intranasal OXT study using the same task revealed that both administration routes produce similar effects on top-down attention control and state anxiety. Overall, our findings suggest that orally administered OXT may have therapeutic utility in disorders with social dysfunction.

## Introduction

The neuropeptide oxytocin (OXT) is an important modulator of social cognition in both animal models and humans (Kendrick, 2000; Kendrick et al., 2018; Quintana et al., 2021) and has been proposed as a therapeutic intervention in disorders with social dysfunction. In particular, treatment with exogenous OXT has been considered in the context of autism spectrum disorder (ASD) (Kendrick et al., 2018). Several clinical trials of daily chronic intranasal OXT treatment have reported modest improvements in social symptoms in children with ASD (Yatawara et al., 2016; Parker et al., 2017), but a recent trial across a wide age range with daily doses of up to 80 IU found no effects (Sikich et al., 2021). There is an ongoing debate in the field, however, as to how OXT administered intranasally produces functional effects and the modulatory influence of dosing strategy or route of administration (Le et al., 2022; Yao and Kendrick, 2022). Notably, recent studies have demonstrated inverted U-curve dose responses to acute OXT (Spengler et al., 2017; Martins et al., 2022) and that chronic daily intranasal doses lead to reduced functional effects (Kou et al., 2020, 2022); 2 recent trials assessing administration of chronic, infrequent (Le et al., 2022) or lower doses (Yamasue et al., 2022) of OXT have reported more robust improvements in ASD symptoms.

Studies investigating the functional effects of acute or chronic OXT treatment in both the clinical and preclinical contexts have mainly used an intranasal administration route, largely guided by initial evidence that the blood-brain barrier (BBB) is relatively impermeable to OXT and that intranasally administered OXT can directly enter the brain through the olfactory and trigeminal nerves (Lee et al., 2020; Quintana et al., 2021; Yeomans et al., 2021). However, intranasal OXT also increases peripheral blood concentrations (Carter, 2014; Zheng and Kendrick, 2021; Yao and Kendrick, 2022) and could produce functional effects either by crossing the BBB after binding to the receptor for advanced glycation end products (RAGE) in endothelial cells (Yamamoto and Higashida, 2020) or by stimulating the vagus nerve via receptors in the heart and gastrointestinal (GI) system (Yao and Kendrick, 2022). Animal model studies have indicated that similar functional effects of OXT can occur following either central or peripheral administration (Ring et al., 2006; Ferris et al., 2015). In humans, 2 initial studies in patients with ASD found positive effects of intravenously administered OXT (Hollander et al., 2003, 2007), although subsequently both negative (Quintana et al., 2016) and positive (Martins et al., 2020) effects of intravenous (IV) OXT have been reported on brain activity.

Recently, we investigated whether an oral (lingual) route of OXT administration in humans can produce functional effects. This route can increase OXT concentrations in the blood and GI system only, with no possibility of direct entry into the brain (De Groot et al., 1995; Kou et al., 2021). Importantly, from a potential therapeutic standpoint in children with ASD, an oral route of chronic administration should be better tolerated than either nasal sprays or IV infusions. The initial results obtained have been encouraging, demonstrating that oral OXT more potently enhanced responses in the brain reward system and amygdala to emotional faces compared with intranasal application (Kou et al., 2021). Furthermore, effects of oral OXT on putamen responses to happy faces were positively associated with increased plasma concentrations of OXT. Oral administration of OXT has also been shown to produce neural and behavioral effects in rodents (Maejima et al., 2020; Tabbaa and Hammock, 2020). Going forward, however, it is important to investigate whether oral OXT can have beneficial functional effects on other aspects of social cognition.

Autism is characterized by marked deficits in social attention (Fletcher-Watson et al., 2009; Kou et al., 2019; Fujioka et al., 2020). Within the framework of social motivation theory, the early-onset social attention impairments in children with ASD are thought to deprive the developing child of social inputs and learning opportunities, which could ultimately lead to diminished social cognition (Chevallier et al., 2012). Intranasally administered OXT has been shown to improve or bias attention to social cues in both typically developing (Ellenbogen et al., 2012; Kendrick et al., 2018; Yao et al., 2018; Xu et al., 2019; Le et al., 2020, 2021) and autistic individuals (Guastella et al., 2010; Yamasue et al., 2020; Huang et al., 2021). In the context of potential therapeutic use, it is therefore important to establish whether orally administered OXT can also enhance attention to social cues.

Against this background, the present study aimed to explore the effect of oral OXT on social attention, including both top-down and bottom-up attention control to social cues, using the same validated emotional antisaccade task we have previously employed to examine the effects of intranasal OXT (Xu et al., 2019). The emotional antisaccade task includes emotional faces (angry, fearful, happy, sad, and neutral) as social stimuli and oval shapes as nonsocial stimuli. Participants are either instructed to look toward the stimuli (prosaccade) or away from them (antisaccade) in this task. During the prosaccade condition, participants focus on the target automatically, which involves “bottom-up” attention processing; during the antisaccade condition, they need to inhibit reflexive orienting toward stimuli by implementing voluntary “top-down” attention control (Munoz and Everling, 2004). In our previous study, we found that intranasal OXT selectively reduced participants’ ability to engage top-down attention control to look away from socially salient stimuli and therefore increased antisaccade errors (Xu et al., 2019). We also found that intranasal OXT decreased post-task state anxiety in line with previous studies showing its anxiolytic effects (Neumann and Landgraf, 2012; Kou et al., 2020). We therefore used the same task to examine whether orally administered OXT produces similar or different effects.

Some previous studies have demonstrated effects of state anxiety on attention control with respect to both antisaccade and prosaccade conditions in this task (Cornwell et al., 2012; Myles et al., 2020). Accumulating evidence indicates that anxiety may contribute to slower antisaccade latencies (considered to reflect performance efficiency) but not to errors (considered performance effectiveness) (Derakshan et al., 2009; Ansari and Derakshan, 2011). In accordance with attention control theory (Eysenck et al., 2007), *performance efficiency* refers to the cognitive resources invested during task performance and is reflected by antisaccade latencies, while *performance effectiveness* refers to the quality of the performance outcome and is indicated by error rates. These findings suggest a specific modulatory role of state anxiety in performance efficiency rather than effectiveness in terms of top-down attention control. Associations between the effects of OXT on anxiety and attention processing in this task have not been examined, however. We therefore additionally investigated whether state anxiety influenced the effects of oral OXT administration on attention control.

To compare the effects of orally and intranasally administered OXT, we compared findings in the current oral dose study with those from our previous intranasal study, where participants received the same OXT dose (Xu et al., 2019). Given increasing evidence for functional effects of exogenous OXT following peripheral changes in concentration, we hypothesized that orally administered OXT would produce similar effects on top-down attentional control as we have previously observed following intranasal administration, particularly with social stimuli. We additionally hypothesized that oral OXT would produce similar decreases in state anxiety. Given previous findings that anxiety does not influence antisaccade errors, we additionally hypothesized that there would be no association between the effects of oral OXT on attention and state anxiety in the context of this task.

## Methods

### Participants

**Figure 1.**
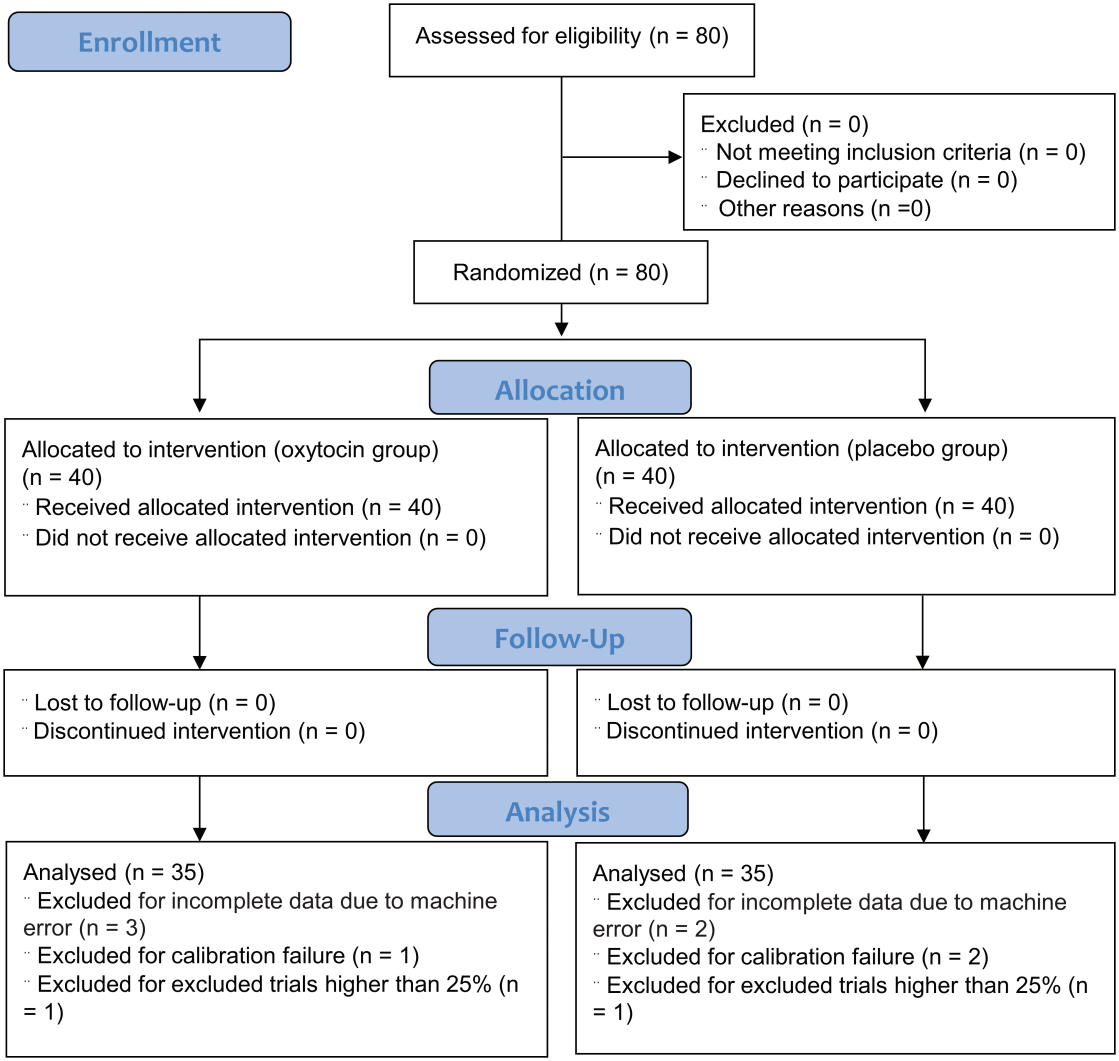
Consolidated Standards of Reporting Trials flow chart.

We recruited 80 healthy right-handed male participants (mean [standard error of the mean (SEM)] age, 22.65 [0.23]) from University of Electronic Science and Technology of China (UESTC). Exclusion criteria were current medication and illicit or licit drug use, including nicotine. The sample size was determined by using G*Power for repeated analysis of variance (ANOVA) (Faul et al., 2009), with an expected medium effect size (*f* = 0.25) and α = .05; 2 treatment groups (OXT and placebo [PLC]) = 2; a specified number of measurements (repeated measures: 2 × 2 = 4); and nonsphericity correction = 1/3. To achieve 80% power, a sample size of 62 is required. Only male participants were included in this study for several reasons: First, to allow comparison with our previous intranasal OXT study, which included only male participants (Xu et al., 2019); second, because of the translational focus on ASD, a disorder that primarily affects male individual; and third, to avoid the influence of the menstrual cycle, which can affect processing of social stimuli in female individuals (Maner and Miller, 2014). All participants were instructed to abstain from consuming alcohol and caffeine during the 24 hours before the experiment. In the placebo-controlled, between-subject design experiment, participants were randomly assigned to receive 24 IU OXT (n = 40; mean [SEM] age, 22.43 [0.28]) or placebo (n = 40; mean [SEM] age, 22.88 [0.36]) (see Figure 1 for a Consolidated Standards of Reporting Trials flow chart of the oral (lingual) spray).

The current study was approved by the local ethics committee of UESTC and preregistered at ClinicalTrials.gov (NCT04493515). All experimental procedures were in accordance with the latest version of the Declaration of Helsinki. All participants provided written informed consent before the experiment and received monetary compensation after completing the experimental tasks.

### Experimental Procedure

To control for potential confounders between different treatment groups, we asked participants to complete validated questionnaires in Chinese before drug treatment, including on mood (Positive and Negative Affect Schedule [PANAS]) (Watson et al., 1988); anxiety (State-Trait Anxiety Inventory [SAI-TAI]) (Spielberger et al., 1983); and other scales measuring depression, autism, childhood experience, cognition, and emotion regulation (see supplementary material). No significant differences were found between the OXT and PLC groups on these questionnaires (all *P* ≥ .08) (Table 1).

**Table 1.** Demographics and Questionnaire Scores for Participants in the Oral PLC and OXT Groups Included in the Final Statistical Analysis

Characteristic	PLC	OXT	*t* Value	*P* Value
Sex, No.	Male, 35	Male, 35	–	–
Age, mean (SEM), y	22.83 (0.41)	22.46 (0.30)	0.73	.47
**Pre-task**				
PANAS, mean (SEM)				
Positive	27.23 (0.92)	28.26 (1.12)	0.71	.48
Negative	15.91 (0.85)	14.89 (0.87)	0.85	.40
STAI, mean (SEM)				
SAI	40.51 (1.53)	40.00 (2.00)	0.21	.84
TAI	40.86 (1.40)	41.97 (1.58)	0.53	.60
LSAS, mean (SEM)				
Avoid	21.23 (1.88)	21.63 (2.22)	0.14	.89
Fear	24.77 (2.15)	23.94 (2.23)	0.27	.79
BDI-II, mean (SEM)	9.29 (1.18)	9.23 (1.64)	0.03	.98
SIAS, mean (SEM)	54.26 (2.13)	50.60 (2.58)	1.10	.28
ASQ, mean (SEM)	21.74 (1.04)	20.46 (1.04)	0.87	.39
CTQ, mean (SEM)	41.60 (1.33)	41.06 (1.45)	0.28	.78
BIS/BAS, mean (SEM)				
BAS–Reward Responsiveness	6.74 (0.24)	6.51 (0.28)	0.62	.54
BAS–Drive	8.11 (0.35)	7.46 (0.31)	1.42	.16
BAS–Fun Seeking	10.43 (0.40)	9.66 (0.38)	1.39	.17
BIS–Behavioral Inhibition	15.80 (0.50)	15.83 (0.47)	0.04	.97
ACS, mean (SEM)				
Failure	5.11 (0.55)	5.77 (0.48)	0.90	.37
Decision	6.31 (0.56)	6.03 (0.49)	0.38	.70
Performance	8.40 (0.31)	8.71 (0.39)	0.64	.53
CERQ, mean (SEM)	47.74 (1.15)	44.71 (1.29)	1.76	.08
**Post-task**				
PANAS, mean (SEM)				
Positive	25.31 (1.20)	25.86 (1.23)	0.32	.75
Negative	14.00 (0.78)	12.94 (0.75)	0.97	.33
SAI, mean (SEM)	39.63 (1.50)	36.46 (1.73)	1.39	.17

Abbreviations: ACS, Action Control Scale; ASQ, Autism Spectrum Quotient; BAS, Behavioral Activation System; BDI-II, Beck Depression Inventory; BIS, Behavioral Inhibition System; CERQ, Cognitive Emotion Regulation Questionnaires; CTQ, Childhood Trauma Questionnaires; LSAS, Liebowitz Social Anxiety Scale; OXT, oxytocin; PANAS, Positive and Negative Affect Schedule; PLC, placebo; SAI, State Anxiety Inventory; SEM, standard error of the mean; SIAS, Social Interaction Anxiety Scale; STAI, State-Trait Anxiety Inventory; TAI, Trait Anxiety Inventory.

**Figure 2.**
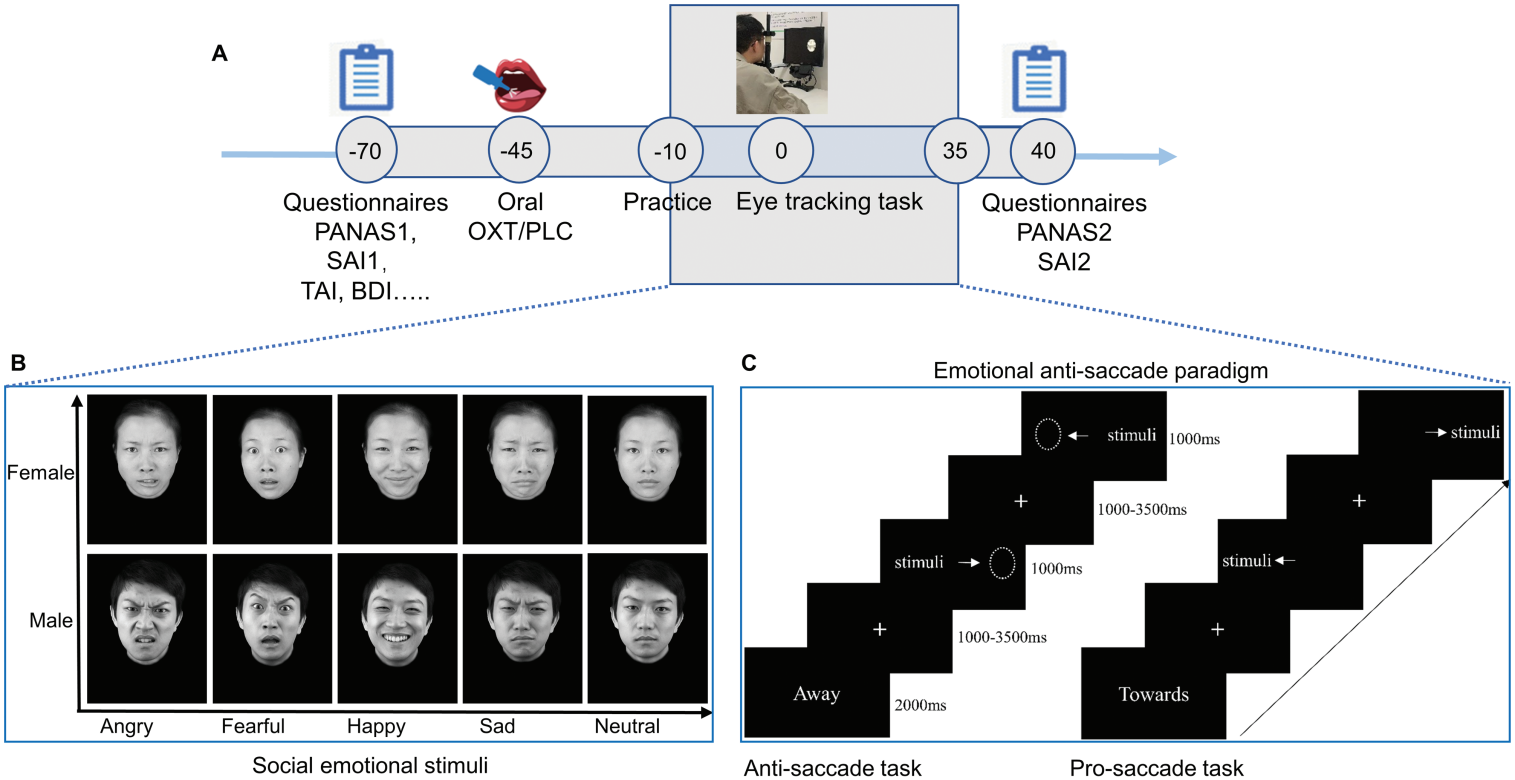
The timing set for experimental procedure (in minutes) and the emotional antisaccade task, including the social-emotional stimuli, were presented (from a Chinese face expression database) (Ma et al., 2022). Abbreviations: PANAS, Positive and Negative Affect Schedule; SAI-TAI, State-Trail Anxiety Inventory.

Next, participants were randomly assigned to receive OXT (Sichuan Meike Pharmaceutical Co Ltd) or placebo (identical spray with the same ingredients but without OXT) administration orally 45 minutes before the antisaccade task (Figure 2A), as in our previous intranasal study (Xu et al., 2019) and in line with the pharmacodynamics of intranasal OXT in humans (Paloyelis et al., 2016; Spengler et al., 2017). Participants were instructed to self-administer the spray 6 times (alternately, 3 puffs on the tongue and 3 puffs under the tongue: 1 puff = 0.1 mL), with 30 seconds between each spray. After each spray, participants were required not to swallow until the next puff to allow more time for absorption by lingual blood vessels. After the whole experiment, participants were asked to guess which treatment they had received: They could not do so better than chance (χ^2^ = 0.98; *P* = .32). Additionally, to examine potential treatment and task effects on mood and state anxiety, we asked participants to complete the PANAS and SAI-TAI before OXT or placebo treatment and after the experimental tasks.

### Experimental Paradigm

The present study used a validated emotional antisaccade paradigm (Chen et al, 2014; Xu et al., 2019) that included 5 social-emotional faces (angry, fearful, happy, sad, and neutral) from 4 female and 4 male actors taken from a published face expression database (Ma et al., 2022) (See Figure 2B) and 8 nonsocial, slightly varying oval shapes. To avoid initial practice effects resulting in increased errors (Smyrnis et al., 2002; Dyckman and McDowell, 2005), all participants performed an initial 32 practice trials (16 anti- and 16 prosaccade trials) before the task. Additionally, to avoid any possible carryover effects of social-emotional stimuli, the nonsocial blocks (2 blocks: 1 antisaccade and 1 prosaccade block), which included 48 trials per block, were always at the beginning of the task, followed by the social blocks (12 blocks: 6 antisaccade and 6 prosaccade blocks), which included 40 trials per block. All blocks and the trials in each block were presented randomly.

Each block began with the 2000-millisecond cue word “towards” or “away.” After the cue, a fixation cross appeared in the center of the monitor, with a mean duration of 1500 milliseconds (jittered time range,1000-3500 ms), and participants were asked to fixate on it. Next, the stimulus was presented on the left or right side of screen for 1000 milliseconds. Participants were required to look toward the stimulus in the “towards” blocks (prosaccade condition) and away in the “away” blocks (antisaccade condition) as accurately and quickly as they could (Figure 2C). The whole task took approximately 35 minutes, with a short rest between blocks.

### Eye Movement Recording and Processing

Eye movement data were recorded in the monocular mode (right eye) using an EyeLink 1000 Plus system (SR Research, Ottawa, Ontario, Canada; 2000-Hz sampling rate), with a screen resolution of 1024 × 768. To fix the standard position and distance from the screen, a chin rest was set 57 cm away from the monitor. At the beginning of each block, a 9-point calibration and validation was performed to ensure eye-tracking data quality. The EyeLink DataViewer 3.1 system (SR Research) preprocessed and exported the raw eye movement data, as it did in our intranasal study (Xu et al., 2019).

In line with previous studies (Reinholdt-Dunne et al., 2012; García-Blanco et al, 2013; Xu et al., 2019), we discarded trials with latencies less than 70 milliseconds or more than 700 milliseconds and saccade velocity lower than 30°/second. In accordance with these criteria, 2 participants were excluded from the final statistical analysis and more than 25% of the trials were deleted. We also excluded data from another 8 participants, including 5 participants with incomplete eye tracking data, because of technical errors; a further 3 participants failed to pass the initial calibration (Reinholdt-Dunne et al., 2012). There was no significant difference in the percentage of excluded trials for the remaining participants between the OXT (n = 35) and PLC groups (n = 35) (PLC mean [SEM], 7.96% [0.85%]; OXT mean [SEM], 9.37% [0.83%]; *t* = 1.18; *P* = .24). An error was defined as a participant making their first saccade in the opposite direction to the 1 they had been instructed to make; we used the number of incorrect trials out of all trials with the same condition (ie, prosaccade or antisaccade) to calculate the error rate for each participant. The saccade response latency for each trial was defined as the duration of the first anti- or prosaccade that occurred after stimuli were presented, and we calculated the averaged correct saccade latencies with the same condition for each participant (Xu et al., 2019). Finally, the mean error rate and latency of correct saccade trials during both anti- and prosaccade conditions served as primary behavioral indexes.

### Statistical Analyses

We examined oral OXT’s effect on attention control, including both top-down attention control and bottom-up attention processing, by means of ANOVAs. First, to determine the effects of oral OXT on attention control for social stimuli, we conducted treatment (OXT/PLC) × condition (social/nonsocial) × task (pro-/antisaccade) mixed ANOVAs on both error rates and latencies. Then, to explore stimulus-specific effects of oral OXT on attention processing, we further conducted treatment × task × stimuli (angry/sad/fearful/happy/neutral/shapes) mixed ANOVAs on error rates and latencies.

Next, we compared the relative effects of OXT administered by oral and intranasal routes on attention control with respect to top-down and bottom-up attention processing with ANOVAs. Mixed ANOVAs on error rates and latencies with treatment (intranasal OXT/oral OXT/intranasal PLC/oral PLC) × condition (social/nonsocial) as factors were conducted on antisaccade and prosaccade tasks, respectively. Additionally, to determine stimulus-specific effects of OXT for different routes on attention processing, treatment × stimuli mixed ANOVAs on error rates and latencies were performed. To control for non–treatment-related variables, we compared the PLC groups from the current and previous intranasal studies with respect to social- and emotion-specific effects on attention control (see supplementary material). The comparison study was preregistered at ClinicalTrials.gov (NCT04815395). Mood and personality traits were also compared between the oral and intranasal OXT administration groups (see supplementary material Table S1). Appropriate Bonferroni-corrected comparisons were employed to disentangle significant main and interaction effects.

## Results

### Effects of Oral OXT on Saccade Error Rates

**Figure 3.**
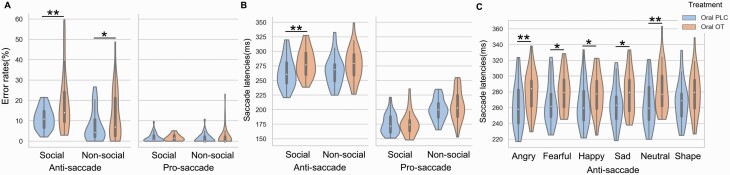
Oral oxytocin’s (OT’s) effect on error rates and latencies. (a) Oral OT increased error rates for both social and nonsocial stimuli (b) but only increased latencies for social stimuli (c) across all emotional faces (effect sizes: angry, Cohen *d *=* *0.62; sad, Cohen *d *=* *0.52; fearful, Cohen *d *=* *0.72; happy, Cohen *d *=* *0.64; neutral, Cohen *d *=* *0.54) but not shapes in the antisaccade task. * and ** denote significant post hoc treatment effects at *P*_*Bonferroni*_ < .05 and *P*_*Bonferroni*_ < .01, respectively.

For error rates, the treatment (OXT/PLC) × condition (social/nonsocial) ×  task (pro-/antisaccade) mixed ANOVA showed a significant main effect of treatment (*F*_(1,68)_ = 6.38; *P* = .014; η^2^p = .09), reflected by higher overall error rates after OXT compared with placebo treatment (PLC mean [SEM], 5.52% [0.90%]; OXT mean [SEM], 8.72% [0.90%]; Cohen *d* = 0.63) (Figure 3A). In addition, a significant treatment × task interaction effect was observed (*F*_(1,68)_ = 8.12; *P* = .006; η^2^p = .11), with post hoc Bonferroni-corrected comparisons showing that compared with placebo treatment, OXT increased error rates in the antisaccade but not prosaccade task (antisaccade PLC mean [SEM], 9.45% [1.56%]; antisaccade OXT mean [SEM], 15.48% [1.56%]; *P* = .008; Cohen *d* = 0.65; prosaccade PLC mean [SEM], 1.59% [0.41%]; prosaccade OXT mean [SEM], 1.95% [0.41%]; *P* = .53). There was no significant treatment × condition (*F*_(1,68)_ = 0.45; *P* = .51) interaction, indicating that OXT had similar effects on errors for social and nonsocial stimuli and no treatment × condition × task interaction (*F*_(1,68)_ = 0.93; *P* = .34). An additional mixed ANOVA, including treatment, task, and stimuli (angry/sad/fearful/ happy/neutral faces and shape), on error rates to explore any stimulus-specific effects did not find a significant treatment × task × stimuli interaction (*F*_(5,340)_ = 0.80; *P* = .51), indicating that OXT did not produce different effects across individual stimuli. Given that participants made few prosaccade errors to influence the data distribution, we carried out an additional analysis following a Box-Cox transformation to control for this effect, and results remained reliable (supplementary results).

### Effects of Oral OXT on Saccade Latencies

For response latencies, we conducted a treatment × condition × task mixed ANOVA. In terms of treatment effects, results showed a significant 3-way interaction among treatment, condition, and task (*F*_(1,68)_ = 6.19; *P* = .015; η^2^p = .08). Post hoc Bonferroni-corrected tests revealed that compared with placebo treatment, oral OXT increased latencies for all social but not nonsocial stimuli in the antisaccade task (social PLC mean [SEM], 263.84 [4.18] ms; social OXT mean [SEM], 279.87 [4.18] ms; *P* = .009; Cohen *d* = 0.65) (nonsocial PLC mean [SEM], 267.56 [4.45] ms; nonsocial OXT mean [SEM], 278.15 [4.45] ms; *P* = .10) (Figure 3B). Effect sizes for individual social stimuli were angry (Cohen *d* = 0.64), sad (Cohen *d* = 0.60), fearful (Cohen *d* = 0.62), happy (Cohen *d* = 0.53), and neutral (Cohen *d* = 0.67) (Figure 3C). There were no other significant interactions involving treatment (all *P* > .069). The additional treatment × task × stimuli mixed ANOVA to explore stimulus-specific effects of OXT on response latency revealed a significant treatment × task × stimuli interaction (*F*_(5,340)_ = 2.50; *P* = .047; η^2^p = .04), although a post hoc Bonferroni-corrected comparison did not reveal any significant differences between specific stimuli in the antisaccade task (all *P* > .30).

### General Effects of Task and Condition on Performance

Our main focus was to analyze treatment-dependent effects in the antisaccade paradigm, but for completeness, we report main and interaction effect task and condition factors in the ANOVAs detailed above (for details, see supplementary material). Results indicate faster prosaccade latencies and increased antisaccade error rates for social compared with nonsocial stimuli, in line with previous findings reporting that social valence modulated attentional processing and also validating the antisaccade paradigm used (Xu et al., 2019; Salvia et al, 2020).

### Effects of Oral OXT on State Anxiety and Mood

**Figure 4.**
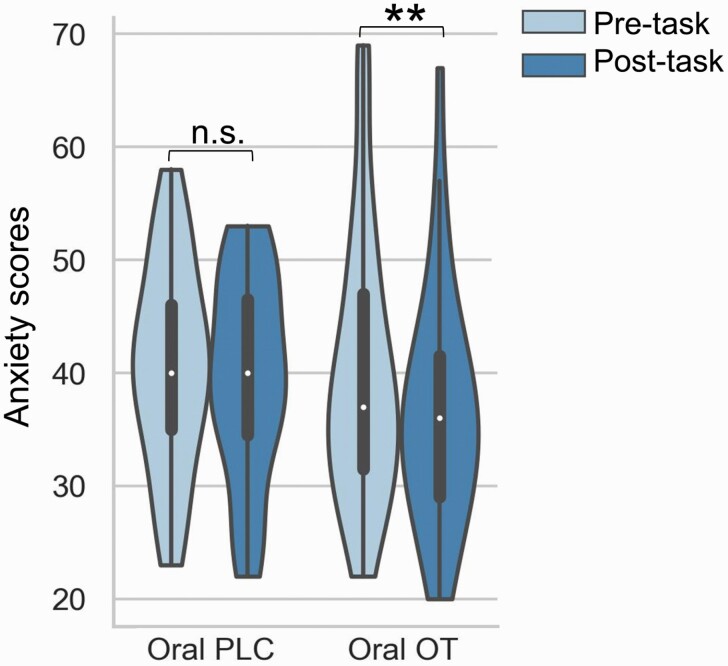
State anxiety scores measured at before and after task in oral the placebo (PLC) and oxytocin (OXT) groups. Abbreviation: n.s., nonsignificant. ** denotes significant difference at *P *<* *.01.

To examine the treatment effect on state anxiety, the repeated ANOVA models on SAI-TAI scores were performed with session (pre-/post-test) as within-subject factor and treatment (oral OXT/oral PLC) as between-subject factor. There was a marginal treatment × time interaction (*F*_(1,68)_ = 2.11; *P* = .15; η^2^p = .03). Exploratory *t* tests were used to further examine changes in the SAI-TAI scores in different groups; these tests indicated that SAI-TAI scores significantly decreased in the oral OXT group (pre-test mean [SEM], 40.00 [1.78]; post-test mean [SEM], 36.46 [1.62]; *P* = .008; Cohen *d* = 0.32) but not in the PLC group (pre-test mean [SEM], 40.51 [1.78]; post-test mean [SEM], 39.63 [1.62]; *P* = .50) (Figure 4). Repeated ANOVA models on PANAS scores revealed no treatment × time interactions for either positive or negative scores (all *P* > .83).

To examine the effects of oral OXT on the association between state anxiety (pre- and post-task SAI-TAI) and attention control (pro- and antisaccade error rates), we conducted Pearson correlations with multiple comparison correction. No significant correlations were observed, however, either in the oral OXT group (all *P* ≥ .079; pre–SAI-TAI prosaccade: *r* = ‒0.15, *P* = .399; pre–SAI-TAI antisaccade: *r* = ‒0.06, *P* = .715; post–SAI-TAI prosaccade: *r* = ‒0.18, *P* = .307; post–SAI-TAI antisaddade: *r* = ‒0.30, *P* = .079) or the PLC groups (all *P* ≥ 0.333; pre–SAI-TAI prosaccade: *r* = ‒0.05, *P* = .785; pre–SAI-TAI antisaccade: *r* = ‒0.01, *P* = .958; post–SAI-TAI prosaccade: *r* = ‒0.17, *P* = .333; post–SAI-TAI antisaccade: *r* = ‒0.06, *P* = .719).

### Comparisons Between the Effects of Intranasal and Oral OXT on Attention Control

**Figure 5.**
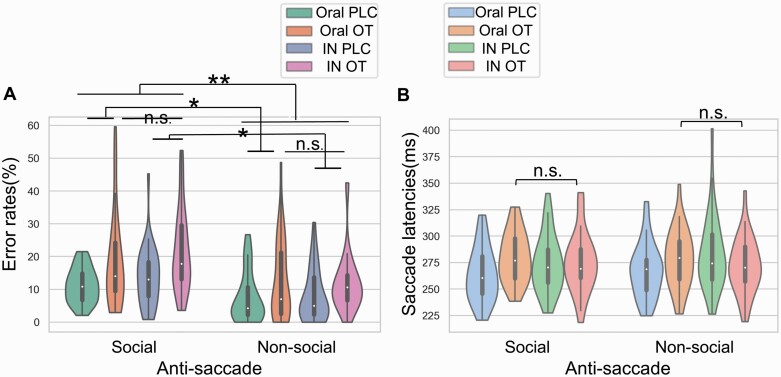
The comparison between intranasal and oral oxytocin’s (OT’s) effect on social attention processing. OT via different routes produced a similar effect on antisaccade errors (a) and latencies (b). Abbreviations: IN, intranasal; n.s., nonsignificant; PLC, placebo. * and ** denote significant post hoc comparisons for the main treatment effect at *P*_*Bonferroni*_ < .05 and *P*_*Bonferroni*_ < .01 respectively.

Given that both our current oral OXT administration and previous intranasal OXT studies (Xu et al., 2019) found significant effects on top-down but not bottom-up attention processing (ie, on antisaccade but not prosaccade performance), we compared the effects of oral and intranasal OXT on antisaccade errors and latencies only. For error rates, the treatment (intranasal OXT/oral OXT/intranasal PLC/oral PLC) ×  condition (social/nonsocial) mixed ANOVA revealed a main effect of treatment (*F*_(3,132)_ = 5.47; *P* = .001; η^2^p = .11). Post hoc tests, however, showed no significant differences between intranasal and oral OXT (*P* > .99); only significant differences between intranasal OXT and intranasal placebo (*P* = .048), intranasal OXT and oral placebo (*P* = .005), and oral OXT and oral placebo (*P* = .028) were found. The interaction effect between treatment and condition was not significant (*F*_(3,132)_ = 1.95; *P* = .12), suggesting that oral and intranasal OXT have similar effects on both social and nonsocial antisaccade errors (Figure 5A). In addition, the mixed ANOVA, including treatment and stimuli (angry, sad, fearful, happy, neutral faces and shapes) on error rates did not reveal a significant treatment × stimuli interaction (*F*_(15,660)_ = 1.41; *P* = .16). This finding indicates that OXT administered by the different routes did not produce any significant differences in antisaccade errors for any specific stimuli.

For anti-saccade response latencies, the mixed ANOVA revealed a treatment × condition interaction effect (*F*_(3,132)_ = 3.13; *P* = .03; η^2^p = .07). Post hoc Bonferroni-corrected comparisons, however, showed no significant differences between intranasal and oral OXT (*P* > .99) (Figure 5B). The additional mixed ANOVA, including treatment and stimuli, also showed a significant treatment × stimuli interaction (*F*_(15,660)_ = 2.34; *P* = .009; η^2^p = .05). Post hoc Bonferroni-corrected comparisons revealed no significant differences between intranasal and oral OXT for individual stimuli and only a significant difference between oral OXT and oral placebo for neutral faces (*P* = .046), although the difference between other emotions approached significance (anxiety: *P* = .07; fearful: *P* = .12; happy: *P* = .26; sad: *P* = .11). These results indicate that in terms of top-down attention control, OXT administered by different routes produces a similar effect on antisaccade latencies.

### Comparison Between the Effects of Oral and Intranasal OXT on State Anxiety

To compare the effects of intranasal and oral OXT on state anxiety, we conducted a group (intranasal OXT/oral OXT/intranasal PLC/oral PLC) ×  SAI-TAI (pre-/post-test) mixed ANOVA on SAI-TAI scores. Neither the interaction effect (*F*_(3,132)_ = 1.91; *P* = .13) nor the main effect of group was significant (*F*_(3,132)_ = 1.42; *P* = .24). A further exploratory post hoc test with Bonferroni correction showed that post-task SAI-TAI scores were significantly decreased in groups receiving oral and intranasal OXT (oral: *P* = .009; intranasal: *P* = .02) but not in the PLC groups (oral: *P* = .51; intranasal: *P* = .77).

### Bayesian Analysis on Nonsignificant Findings

To further examine the nonsignificant hypothesis that oral and intranasal OXT have similar effects on top-down attentional control and anxiety, Bayesian analysis was conducted using JASP, version 0.14.1.0, statistical software (The JASP Team, University of Amsterdam, Amsterdam, The Netherlands). Results showed a moderate fit for errors and state anxiety but only an anecdotal fit for response latencies (for details, see supplementary material).

## Discussion

Overall, the current pharmacologic study using an emotional antisaccade eye tracking paradigm revealed that orally administered OXT increased antisaccade error rates for both social and nonsocial stimuli but only increased response latencies for all social stimuli, suggesting that oral OXT may particularly reduce performance efficiency with respect to top-down social attention control. Oral OXT also reduced state anxiety scores, indicating an anxiolytic effect, although there was no association between state anxiety scores and task performance. An additional comparison for the effects of oral vs intranasal OXT administration revealed no significant differences between routes of administration either on the antisaccade task errors and response latencies or on anxiolytic effects. Together, these findings suggest that oral OXT may produce a similar therapeutic effect as intranasal OXT, especially in disorders with social attention deficits.

In the current study, our main finding was that orally administered OXT increased errors for both social (angry, fearful, happy, sad, and neutral emotion faces) and nonsocial (oval shapes) stimuli in the antisaccade task but only increased saccade response latencies for social-emotional faces. This finding suggests that oral OXT treatment has a general effect on top-down attention control in terms of performance effectiveness (errors) but a more selective effect on performance efficiency (saccade response latencies) for social stimuli. Based on the framework of attention control theory (Eysenck et al., 2007), the results indicate that following oral OXT relative to placebo treatment, participants need to dedicate more effort and attentional resources to shift attention away from stimuli, particularly social stimuli. In addition, enhanced error rates for stimuli across all categories may reflect increased arousal to both social and nonsocial stimuli after oral OXT treatment. In a previous study, we found that lingual OXT increased arousal responses to social stimuli (face expressions) (Kou et al., 2021), and another study using intranasal OXT administration reported increased arousal (indicated by increased pupil dilation) to both social and nonsocial stimuli (Eckstein et al., 2019).

Our other main finding in the present study was that orally administered OXT produced effects similar to intranasal OXT on top-down social attention in terms of both increased antisaccade errors and longer saccade response times during presentation of stimuli. We used Bayes to test the robustness of the nonsignificant difference between the routes of administration, and a Bayes factor greater than 3 was found for analysis of antisaccade errors, indicating moderate evidence for the null model of the nonsignificant difference (Jeffreys, 1998). Although the previous intranasal OXT study did report some evidence for a social-specific effects of treatment, the evidence was only marginal, which may explain the lack of evidence for a statistical difference between the effects of oral and intranasal routes. For the antisaccade response latency analysis, however, we found only anecdotal evidence to support the null hypothesis, suggesting that there may be some differential effect of the 2 routes of administration. Indeed, only oral OXT significantly increased response times for the social stimuli.

Similar to intranasal OXT administration, oral OXT reduced post-task state anxiety scores, indicating an anxiolytic effect. Again, the magnitude of effects the 2 routes of administration produced were similar, and the bayesian analysis revealed a moderate Bayes factor greater than 3, supporting the null hypothesis. These findings are in line with several previous studies reporting anxiolytic effects of intranasal OXT, but we found no evidence to support a significant association between performance on the attention task used and state anxiety with either OXT or placebo treatment. Thus, it seems unlikely that in the context of this specific paradigm, effects of OXT on top-down attention were influenced by its anxiolytic effects. Some previous studies have shown that increased rather than decreased anxiety can decrease top-down attention (Derakshan et al., 2009; Ansari and Derakshan, 2011) or increase autonomic bottom-up attention processing (Cornwell et al., 2012), while others have reported enhanced top-down attention control indicative of faster antisaccade latency times in anxious compared with control individuals (Cardinale et al., 2019) and shown that anxiety can facilitate individuals’ cognitive performance (Grillon et al., 2016). Unlike the majority of the above studies reporting effects of anxiety on top-down attention, we did not only include participants with high trait anxiety; this decision may have resulted in a lack of association between state anxiety and attention performance. Also, reduced state anxiety scores for participants on OXT treatment may not have been sufficiently high to significantly influence performance.

In terms of the ongoing debate concerning how intranasally administered OXT may act to modulate neural and behavioral functions, our findings provide further support for the importance of increased OXT concentrations in the peripheral vascular system, producing neural changes either through RAGE-mediated transport across the BBB or through vagal stimulation (see Yao and Kendrick, 2022). We required participants to retain the OXT lingual spray in their mouths between repeated sprays to maximize the potential for oromucosal absorption into peripheral circulation. Although it is possible that OXT could additionally be absorbed from the GI system into the blood through RAGE (Higashida et al., 2017), pepsin in the acidic conditions of the stomach is likely to break down the peptide quickly. Indeed, a study in rodents has shown that oral administration of OXT in capsule form showed markedly enhanced peripheral concentrations only after preadministration of the proton pump inhibitor (PPI) omeprazole (Maejima et al., 2020). These findings do not rule out the additional contributions of direct entry of OXT into the brain through the olfactory and trigeminal nerves following intranasal administration, but they provide increased support for this not being the only route. Interestingly, however, the current findings, together with those of our previous study (Kou et al., 2021) and from another group (Martins et al., 2020), raise the possibility of administration route–dependent functional effects that require further exploration and may ultimately influence therapeutic intervention strategy. Although the temporal profiles of plasma concentration changes following 24 IU oral or intranasal administration of OXT are broadly similar, the overall increases following oral administration are significantly smaller than those found after intranasal administration (Kou et al., 2021), raising the possibility that route-dependent functional effects may be influenced by dose. Thus, at this stage it is possible there may be interactions between OXT dose (both concentration and frequency) and route of administration that determine functional effects.

The effects of oral administration of OXT on top-down social attention processing are similar to those we have previously reported after intranasal administration of vasopressin (Zhuang et al., 2021). This finding suggests that both peptides have facilitatory effects on social attention and that both may act to enhance the salience of social stimuli, as has been proposed for OXT (Shamay-Tsoory and Abu-Akel, 2016). The findings may also help explain why both peptides have been shown to improve social responsivity in children with ASD (Yatawara et al., 2016; Parker et al., 2017, 2019; Le et al., 2022). There is the possibility of cross-talk between receptors because both peptides can stimulate each other’s receptors to some extent (Neumann and Landgraf, 2012). With at least a 10-fold reduction in affinity for the other peptide’s receptors, however, it seems more likely that both produce enhanced social attention by acting on their own receptors.

We acknowledge several limitations in the current study. First, only male participants were included in the present study to be in line with our intranasal OXT study (Xu et al., 2019) and given a translational focus on autism. Some previous studies in rodents have reported sex-specific behavioral and neural effects of peripherally administered OXT (Dumais et al, 2017; Tabbaa and Hammock, 2020), and some studies in humans with intranasal OXT administration have also reported sex-dependent effects (Gao et al., 2016; Luo et al., 2017), although not for social attention paradigms. Future studies will be needed to explore oral OXT’s effect on attention control in both male and female participants.

Second, in line with previous studies, oval shapes served as nonsocial stimuli but are less complex than emotional faces. Future studies could consider using objects or natural scenes as nonsocial control stimuli to match the complexity of faces. Third, given that the PANAS and SAI-TAI questionnaires were measured only before oral treatment and after tasks, it cannot be determined whether the observed anxiolytic effect in the oral OXT group was the result of OXT treatment per se or a combination of treatment and task. For example, a previous study has shown that task difficulty could affect individuals’ state anxiety level (O’Neil et al., 1969). The number of errors and longer response times among participants in the antisaccade condition during the task do suggest that participants found it difficult.

Fourth, note that the total sample size (N = 151 [intranasal study: n = 71; oral study: n = 80) is sufficient to achieve 80% power for a medium effect size (f = 0.25) in the comparison study. Treatment groups, however, were only randomly assigned within the 2 independent experiments and are not a priori matched for the comparison study, although this approach is in line with our previous studies comparing the effects of OXT and vasopressin in this same task (Zhuang et al., 2021) and also OXT’s effect on emotional processing through oral and intranasal routes (Kou et al., 2021). Treatment groups in both studies were also initially matched for several potential confounding demographic, mood, state anxiety, and personality trait variables.

In summary, the current study used a validated emotional antisaccade paradigm that can measure treatment effects on both top-down and bottom-up attentional processing and revealed that in terms of top-down attention control, oral OXT decreased performance effectiveness across all stimuli but selectively modulated performance efficiency for social stimuli. Comparison of the effects of oral OXT with those we have previously reported for intranasal OXT revealed that OXT through both routes produced similar effects on top-down attention and state anxiety. Overall, these findings suggest that some key functional effects of exogenous OXT administration involve an influence on the brain through the peptide being transported from the blood across the BBB or by vagally mediated effects. The findings also suggest that OXT administered by an oral route may be useful in a therapeutic context, particularly for children, where intranasal administration may be less well tolerated.

## Supplementary Material

pyac059_suppl_Supplementary_Material

## References

[CIT0001] Ansari TL , Derakshan N (2011) The neural correlates of impaired inhibitory control in anxiety. Neuropsychologia 49:1146–1153. 21241717 10.1016/j.neuropsychologia.2011.01.019

[CIT0002] Cardinale EM , Subar AR, Brotman MA, Leibenluft E, Kircanski K, Pine DS (2019) Inhibitory control and emotion dysregulation: a framework for research on anxiety. Dev Psychopathol 31:859–869. 30968800 10.1017/S0954579419000300 PMC7439288

[CIT0003] Carter CS (2014) Oxytocin pathways and the evolution of human behavior. Annu Rev Psychol 65:17–39. 24050183 10.1146/annurev-psych-010213-115110

[CIT0004] Chen NT , Clarke PJ, Watson TL, MacLeod C, Guastella AJ (2014) Biased saccadic responses to emotional stimuli in anxiety: an antisaccade study. PLoS One 9:e86474. 24523861 10.1371/journal.pone.0086474 PMC3921140

[CIT0005] Chevallier C , Kohls G, Troiani V, Brodkin ES, Schultz RT (2012) The social motivation theory of autism. Trends Cogn Sci 16:231–239. 22425667 10.1016/j.tics.2012.02.007 PMC3329932

[CIT0006] Cornwell BR , Mueller SC, Kaplan R, Grillon C, Ernst M (2012) Anxiety, a benefit and detriment to cognition: behavioral and magnetoencephalographic evidence from a mixed-saccade task. Brain Cogn 78:257–267. 22289426 10.1016/j.bandc.2012.01.002 PMC3448553

[CIT0007] De Groot AN , Vree TB, Hekster YA, Pesman GJ, Sweep FC, Van Dongen PJ, Van Roosmalen J (1995) Bioavailability and pharmacokinetics of sublingual oxytocin in male volunteers. J Pharm Pharmacol 47:571–575. 8568623 10.1111/j.2042-7158.1995.tb06716.x

[CIT0008] Derakshan N , Ansari TL, Hansard M, Shoker L, Eysenck MW (2009) Anxiety, inhibition, efficiency, and effectiveness: an investigation using the antisaccade task. Exp Psychol 56:48–55. 19261578 10.1027/1618-3169.56.1.48

[CIT0009] Dumais KM , Kulkarni PP, Ferris CF, Veenema AH (2017) Sex differences in neural activation following different routes of oxytocin administration in awake adult rats. Psychoneuroendocrinology 81:52–62. 28412582 10.1016/j.psyneuen.2017.04.003 PMC5497485

[CIT0010] Dyckman KA , McDowell JE (2005) Behavioral plasticity of antisaccade performance following daily practice. Exp Brain Res 162:63–69. 15551081 10.1007/s00221-004-2105-9

[CIT0011] Eckstein M , Bamert V, Stephens S, Wallen K, Young LJ, Ehlert U, Ditzen B (2019) Oxytocin increases eye-gaze towards novel social and non-social stimuli. Soc Neurosci 14:594–607. 30378456 10.1080/17470919.2018.1542341 PMC6494727

[CIT0012] Ellenbogen MA , Linnen AM, Grumet R, Cardoso C, Joober R (2012) The acute effects of intranasal oxytocin on automatic and effortful attentional shifting to emotional faces. Psychophysiology 49:128–137. 22092248 10.1111/j.1469-8986.2011.01278.x

[CIT0013] Eysenck MW , Derakshan N, Santos R, Calvo MG (2007) Anxiety and cognitive performance: attentional control theory. Emotion 7:336–353. 17516812 10.1037/1528-3542.7.2.336

[CIT0014] Faul F , Erdfelder E, Buchner A, Lang AG (2009) Statistical power analyses using G* Power 3.1: tests for correlation and regression analyses. Behav Res Methods 41:1149–1160. 19897823 10.3758/BRM.41.4.1149

[CIT0015] Ferris CF , Yee JR, Kenkel WM, Dumais KM, Moore K, Veenema AH, Kulkarni P, Perkybile AM, Carter CS (2015) Distinct BOLD activation profiles following central and peripheral oxytocin administration in awake rats. Front Behav Neurosci 9:245. 26441574 10.3389/fnbeh.2015.00245 PMC4585275

[CIT0016] Fletcher-Watson S , Leekam SR, Benson V, Frank MC, Findlay JM (2009) Eye-movements reveal attention to social information in autism spectrum disorder. Neuropsychologia 47:248–257. 18706434 10.1016/j.neuropsychologia.2008.07.016

[CIT0017] Fujioka T , et al. (2020) Developmental changes in attention to social information from childhood to adolescence in autism spectrum disorders: a comparative study. Mol Autism 11:24. 32272970 10.1186/s13229-020-00321-w PMC7146883

[CIT0018] Gao S , Becker B, Luo L, Geng Y, Zhao W, Yin Y, Hu J, Gao Z, Gong Q, Hurlemann R, Yao D, Kendrick KM (2016) Oxytocin, the peptide that bonds the sexes also divides them. Proc Natl Acad Sci U S A 113:7650–7654. 27325780 10.1073/pnas.1602620113 PMC4941426

[CIT0019] García-Blanco AC , Perea M, Salmerón L (2013) Attention orienting and inhibitory control across the different mood states in bipolar disorder: an emotional antisaccade task. Biol Psychol 94:556–561. 24161800 10.1016/j.biopsycho.2013.10.005

[CIT0020] Grillon C , Robinson OJ, Mathur A, Ernst M (2016) Effect of attention control on sustained attention during induced anxiety. Cogn Emot 30:700–712. 25899613 10.1080/02699931.2015.1024614 PMC4618278

[CIT0021] Guastella AJ , Einfeld SL, Gray KM, Rinehart NJ, Tonge BJ, Lambert TJ, Hickie IB (2010) Intranasal oxytocin improves emotion recognition for youth with autism spectrum disorders. Biol Psychiatry 67:692–694. 19897177 10.1016/j.biopsych.2009.09.020

[CIT0022] Higashida H , Furuhara K, Yamauchi AM, Deguchi K, Harashima A, Munesue S, Lopatina O, Gerasimenko M, Salmina AB, Zhang JS, Kodama H, Kuroda H, Tsuji C, Yamamoto H, Yamamoto Y (2017) Intestinal transepithelial permeability of oxytocin into the blood is dependent on the receptor for advanced glycation end products in mice. Sci Rep 7:7883. 28801574 10.1038/s41598-017-07949-4 PMC5554167

[CIT0023] Hollander E , Novotny S, Hanratty M, Yaffe R, DeCaria CM, Aronowitz BR, Mosovich S (2003) Oxytocin infusion reduces repetitive behaviors in adults with autistic and Asperger’s disorders. Neuropsychopharmacology 28:193–198. 12496956 10.1038/sj.npp.1300021

[CIT0024] Hollander E , Bartz J, Chaplin W, Phillips A, Sumner J, Soorya L, Anagnostou E, Wasserman S (2007) Oxytocin increases retention of social cognition in autism. Biol Psychiatry 61:498–503. 16904652 10.1016/j.biopsych.2006.05.030

[CIT0025] Huang Y , Huang X, Ebstein RP, Yu R (2021) Intranasal oxytocin in the treatment of autism spectrum disorders: a multilevel meta-analysis. Neurosci Biobehav Rev 122:18–27. 33400920 10.1016/j.neubiorev.2020.12.028

[CIT0026] Jeffreys H (1998) The theory of probability, 3rd ed. Oxford: Oxford UP.

[CIT0027] Kendrick KM (2000) Oxytocin, motherhood and bonding. Exp Physiol 85 Spec No:111s–124s. 10795913 10.1111/j.1469-445x.2000.tb00014.x

[CIT0028] Kendrick KM , Guastella AJ, Becker B (2018) Overview of human oxytocin research. Curr Top Behav Neurosci 35:321–348. 28864976 10.1007/7854_2017_19

[CIT0029] Kou J , Le J, Fu M, Lan C, Chen Z, Li Q, Zhao W, Xu L, Becker B, Kendrick KM (2019) Comparison of three different eye-tracking tasks for distinguishing autistic from typically developing children and autistic symptom severity. Autism Res 12:1529–1540. 31369217 10.1002/aur.2174

[CIT0030] Kou J , Zhang Y, Zhou F, Sindermann C, Montag C, Becker B, Kendrick KM (2020) A randomized trial shows dose-frequency and genotype may determine the therapeutic efficacy of intranasal oxytocin. Psychol Med 4:1–10. 10.1017/S0033291720003803 33272333

[CIT0031] Kou J , Lan C, Zhang Y, Wang Q, Zhou F, Zhao Z, Montag C, Yao S, Becker B, Kendrick KM (2021) In the nose or on the tongue? Contrasting motivational effects of oral and intranasal oxytocin on arousal and reward during social processing. Transl Psychiatry 11:94. 33542175 10.1038/s41398-021-01241-w PMC7862637

[CIT0032] Kou J , Zhang Y, Zhou F, Gao Z, Yao S, Zhao W, Li H, Lei Y, Gao S, Kendrick KM, Becker B (2022) Anxiolytic effects of chronic intranasal oxytocin on neural responses to threat are dose-frequency dependent. Psychother Psychosom 91:253–264. 35086102 10.1159/000521348

[CIT0033] Le J , Kou J, Zhao W, Fu M, Zhang Y, Becker B, Kendrick KM (2020) Oxytocin biases eye-gaze to dynamic and static social images and the eyes of fearful faces: associations with trait autism. Transl Psychiatry 10:142. 32398642 10.1038/s41398-020-0830-x PMC7217872

[CIT0034] Le J , Zhao W, Kou J, Fu M, Zhang Y, Becker B, Kendrick KM (2021) Oxytocin facilitates socially directed attention. Psychophysiology 58:e13852. 34032304 10.1111/psyp.13852

[CIT0035] Le J , Zhang L, Zhao W, Zhu S, Lan C, Kou J, Zhang Q, Zhang Y, Li Q, Chen Z, Fu M, Montag C, Zhang R, Yang W, Becker B, Kendrick KM (2022) Infrequent intranasal oxytocin followed by positive social interaction improves symptoms in autistic children: a pilot randomized clinical trial. Psychother Psychosom 91:335–347. 35545057 10.1159/000524543

[CIT0036] Lee MR , Shnitko TA, Blue SW, Kaucher AV, Winchell AJ, Erikson DW, Grant KA, Leggio L (2020) Labeled oxytocin administered via the intranasal route reaches the brain in rhesus macaques. Nat Commun 11:2783. 32494001 10.1038/s41467-020-15942-1 PMC7270110

[CIT0037] Luo L , Becker B, Geng Y, Zhao Z, Gao S, Zhao W, Yao S, Zheng X, Ma X, Gao Z, Hu J, Kendrick KM (2017) Sex-dependent neural effect of oxytocin during subliminal processing of negative emotion faces. Neuroimage 162:127–137. 28877512 10.1016/j.neuroimage.2017.08.079

[CIT0038] Ma X , Fu M, Zhang X, Song X, Becker B, Wu R, Xu X, Gao Z, Kendrick K, Zhao W (2022) Own race eye-gaze bias for all emotional faces but accuracy bias only for sad expressions. Front Neurosci 16:852484. 35645716 10.3389/fnins.2022.852484 PMC9133890

[CIT0039] Maejima Y , Horita S, Otsuka A, Hidema S, Nishimori K, Shimomura K (2020) Oral oxytocin delivery with proton pump inhibitor pretreatment decreases food intake. Peptides 128:170312. 32298773 10.1016/j.peptides.2020.170312

[CIT0040] Maner JK , Miller SL (2014) Hormones and social monitoring: menstrual cycle shifts in progesterone underlie women’s sensitivity to social information. Evol Hum Behav 35:9–16.

[CIT0041] Martins DA , Mazibuko N, Zelaya F, Vasilakopoulou S, Loveridge J, Oates A, Maltezos S, Mehta M, Wastling S, Howard M, McAlonan G, Murphy D, Williams SCR, Fotopoulou A, Schuschnig U, Paloyelis Y (2020) Effects of route of administration on oxytocin-induced changes in regional cerebral blood flow in humans. Nat Commun 11:1160. 32127545 10.1038/s41467-020-14845-5 PMC7054359

[CIT0042] Martins D , Brodmann K, Veronese M, Dipasquale O, Mazibuko N, Schuschnig U, Zelaya F, Fotopoulou A, Paloyelis Y (2022) “Less is more”: a dose-response account of intranasal oxytocin pharmacodynamics in the human brain. Prog Neurobiol 211:102239. 35122880 10.1016/j.pneurobio.2022.102239

[CIT0043] Munoz DP , Everling S (2004) Look away: the anti-saccade task and the voluntary control of eye movement. Nat Rev Neurosci 5:218–228. 14976521 10.1038/nrn1345

[CIT0044] Myles O , Grafton B, MacLeod C (2020) Anxiety & inhibition: dissociating the involvement of state and trait anxiety in inhibitory control deficits observed on the anti-saccade task. Cogn Emot 34:1746–1752. 32746726 10.1080/02699931.2020.1802229

[CIT0045] Neumann ID , Landgraf R (2012) Balance of brain oxytocin and vasopressin: implications for anxiety, depression, and social behaviors. Trends Neurosci 35:649–659. 22974560 10.1016/j.tins.2012.08.004

[CIT0046] O’Neil HF Jr , Spielberger CD, Hansen DN (1969) Effects of state anxiety and task difficulty on computer-assisted learning. J Educ Psychol 60:343–350. 5364288 10.1037/h0028323

[CIT0047] Paloyelis Y , Doyle OM, Zelaya FO, Maltezos S, Williams SC, Fotopoulou A, Howard MA (2016) A spatiotemporal profile of in vivo cerebral blood flow changes following intranasal oxytocin in humans. Biol Psychiatry 79:693–705. 25499958 10.1016/j.biopsych.2014.10.005

[CIT0048] Parker KJ , Oztan O, Libove RA, Sumiyoshi RD, Jackson LP, Karhson DS, Summers JE, Hinman KE, Motonaga KS, Phillips JM, Carson DS, Garner JP, Hardan AY (2017) Intranasal oxytocin treatment for social deficits and biomarkers of response in children with autism. Proc Natl Acad Sci U S A 114:8119–8124. 28696286 10.1073/pnas.1705521114 PMC5544319

[CIT0049] Parker KJ , Oztan O, Libove RA, Mohsin N, Karhson DS, Sumiyoshi RD, Summers JE, Hinman KE, Motonaga KS, Phillips JM, Carson DS, Fung LK, Garner JP, Hardan AY (2019) A randomized placebo-controlled pilot trial shows that intranasal vasopressin improves social deficits in children with autism. Sci Transl Med 11:eaau7356. 31043522 10.1126/scitranslmed.aau7356 PMC6716148

[CIT0050] Quintana DS , Westlye LT, Alnæs D, Rustan ØG, Kaufmann T, Smerud KT, Mahmoud RA, Djupesland PG, Andreassen OA (2016) Low dose intranasal oxytocin delivered with Breath Powered device dampens amygdala response to emotional stimuli: a peripheral effect-controlled within-subjects randomized dose-response fMRI trial. Psychoneuroendocrinology 69:180–188. 27107209 10.1016/j.psyneuen.2016.04.010

[CIT0051] Quintana DS , Lischke A, Grace S, Scheele D, Ma Y, Becker B (2021) Advances in the field of intranasal oxytocin research: lessons learned and future directions for clinical research. Mol Psychiatry 26:80–91. 32807845 10.1038/s41380-020-00864-7 PMC7815514

[CIT0052] Reinholdt-Dunne ML , Mogg K, Benson V, Bradley BP, Hardin MG, Liversedge SP, Pine DS, Ernst M (2012) Anxiety and selective attention to angry faces: an antisaccade study. J Cogn Psychol 24:54–65.

[CIT0053] Ring RH , Malberg JE, Potestio L, Ping J, Boikess S, Luo B, Schechter LE, Rizzo S, Rahman Z, Rosenzweig-Lipson S (2006) Anxiolytic-like activity of oxytocin in male mice: behavioral and autonomic evidence, therapeutic implications. Psychopharmacology (Berl) 185:218–225. 16418825 10.1007/s00213-005-0293-z

[CIT0054] Salvia E , Harvey M, Nazarian B, Grosbras MH (2020) Social perception drives eye-movement related brain activity: evidence from pro-and anti-saccades to faces. Neuropsychologia 139:107360. 31982482 10.1016/j.neuropsychologia.2020.107360

[CIT0055] Shamay-Tsoory SG , Abu-Akel A (2016) The social salience hypothesis of oxytocin. Biol Psychiatry 79:194–202. 26321019 10.1016/j.biopsych.2015.07.020

[CIT0056] Sikich L , et al (2021) Intranasal oxytocin in children and adolescents with autism spectrum disorder. N Engl J Med 385:1462–1473. 34644471 10.1056/NEJMoa2103583 PMC9701092

[CIT0057] Smyrnis N , Evdokimidis I, Stefanis NC, Constantinidis TS, Avramopoulos D, Theleritis C, Paximadis C, Efstratiadis C, Kastrinakis G, Stefanis CN (2002) The antisaccade task in a sample of 2,006 young males II. Effects of task parameters. Exp Brain Res 147:53–63. 12373369 10.1007/s00221-002-1207-5

[CIT0058] Spengler FB , Schultz J, Scheele D, Essel M, Maier W, Heinrichs M, Hurlemann R (2017) Kinetics and dose dependency of intranasal oxytocin effects on amygdala reactivity. Biol Psychiatry 82:885–894. 28629540 10.1016/j.biopsych.2017.04.015

[CIT0059] Spielberger CD , Gorsuch RL, Lushene R, Vagg PR, Jacobs GA (1983) Manual for the state-trait anxiety inventory (STAI). San Diego, CA: Mindgarden.

[CIT0060] Tabbaa M , Hammock EAD (2020) Orally administered oxytocin alters brain activation and behaviors of pre-weaning mice. Horm Behav 118:104613. 31654673 10.1016/j.yhbeh.2019.104613 PMC7015803

[CIT0061] Watson D , Clark LA, Carey G (1988) Positive and negative affectivity and their relation to anxiety and depressive disorders. J Abnorm Psychol 97:346–353. 3192830 10.1037//0021-843x.97.3.346

[CIT0062] Xu X , Li J, Chen Z, Kendrick KM, Becker B (2019) Oxytocin reduces top-down control of attention by increasing bottom-up attention allocation to social but not non-social stimuli—a randomized controlled trial. Psychoneuroendocrinology 108:62–69. 31229634 10.1016/j.psyneuen.2019.06.004

[CIT0063] Yamamoto Y , Higashida H (2020) RAGE regulates oxytocin transport into the brain. Commun Biol 3:1–4. 32054984 10.1038/s42003-020-0799-2 PMC7018824

[CIT0064] Yamasue H , et al. (2020) Effect of intranasal oxytocin on the core social symptoms of autism spectrum disorder: a randomized clinical trial. Mol Psychiatry 25:1849–1858. 29955161 10.1038/s41380-018-0097-2

[CIT0065] Yamasue H , et al. (2022) Effect of a novel nasal oxytocin spray with enhanced bioavailability on autism. Brain 145:490–499. 35067719 10.1093/brain/awab291

[CIT0066] Yao S , Kendrick KM (2022) Effects of intranasal administration of oxytocin and vasopressin on social cognition and potential routes and mechanisms of action. Pharmaceutics 14:323. 35214056 10.3390/pharmaceutics14020323 PMC8874551

[CIT0067] Yao S , Becker B, Zhao W, Zhao Z, Kou J, Ma X, Geng Y, Ren P, Kendrick KM (2018) Oxytocin modulates attention switching between interoceptive signals and external social cues. Neuropsychopharmacology 43:294–301. 28836577 10.1038/npp.2017.189 PMC5729568

[CIT0068] Yatawara CJ , Einfeld SL, Hickie IB, Davenport TA, Guastella AJ (2016) The effect of oxytocin nasal spray on social interaction deficits observed in young children with autism: a randomized clinical crossover trial. Mol Psychiatry 21:1225–1231. 26503762 10.1038/mp.2015.162 PMC4995545

[CIT0069] Yeomans DC , Hanson LR, Carson DS, Tunstall BJ, Lee MR, Tzabazis AZ, Jacobs D, Frey WH 2nd (2021) Nasal oxytocin for the treatment of psychiatric disorders and pain: achieving meaningful brain concentrations. Transl Psychiatry 11:388. 34247185 10.1038/s41398-021-01511-7 PMC8272715

[CIT0070] Zheng X , Kendrick KM (2021) Neural and molecular contributions to pathological jealousy and a potential therapeutic role for intranasal oxytocin. Front Pharmacol 12:652473. 33959017 10.3389/fphar.2021.652473 PMC8094533

[CIT0071] Zhuang Q , Zheng X, Becker B, Lei W, Xu X, Kendrick KM (2021) Intranasal vasopressin like oxytocin increases social attention by influencing top-down control, but additionally enhances bottom-up control. Psychoneuroendocrinology 133:105412. 34537624 10.1016/j.psyneuen.2021.105412

