# Supplementary material for: Oral Administration of Oxytocin, Like Intranasal Administration, Decreases Top-Down Social Attention
Source: Int J Neuropsychopharmacol. 2022 Sep 2;25(11):912–23. doi: 10.1093/ijnp/pyac059 (PMC9670742; doi:10.1093/ijnp/pyac059)
Supplement: pyac059_suppl_Supplementary_Material [file pyac059_suppl_supplementary_material.docx]

SUPPLEMENTARY MATERIAL

**Oral, similar to intranasal, administration of oxytocin decreases top-down social attention**

Qian Zhuang^#^, Xiaoxiao Zheng^#^, Shuxia Yao, Weihua Zhao, Benjamin Becker, Xiaolei Xu, Keith M. Kendrick*

**Comparisons between intranasal and oral PLC groups**

To control for non-treatment related factors, PLC groups from the current study and previous intranasal OXT study (PLC/PLC2) were compared with respect to the social- and emotion-specific effects on attentional control.

For error rates, the 2 (group: PLC/PLC2) * 2 (condition: social/non-social) * 2 (task: pro- /anti-saccade) mixed ANOVA was conducted to examine the social effects on attention processing between two PLC groups. No group related main effect or interaction effects were observed (all *ps* ≥ 0.20). Results found a main effect of condition (F_1, 66_ = 25.03, *p* < 0.001, η^2^p = 0.28) and task (F_1, 66_ = 130.52, *p* < 0.001, η^2^p = 0.66) and also a significant interaction effect between them (F_1, 66_ = 16.52, *p* < 0.001, η^2^p = 0.20). The 2 (group: PLC/PLC2) * 2 (task: pro-/anti-saccade) * 6 (stimuli: angry/sad/fearful/happy/neutral/shapes) mixed ANOVA on error rate showed no group related main effect or interaction effects (all *ps* ≥ 0.10).

For latencies, the 2 (group: PLC/PLC2) * 2 (condition: social/non-social) * 2 (task: pro-/anti-saccade) mixed ANOVA was performed to examine the social effects on attention processing between two PLC groups. Results showed a significant main effect of task (F_1, 66_ = 725.72, *p* < 0.001, η^2^p = 0.92) and condition (F_1, 66_ = 107.94, *p* < 0.001, η^2^p = 0.62) and a significant interaction between them (F_1, 66_ = 45.35, *p* < 0.001, η^2^p = 0.41). No group related main effect or interaction effects were observed (all *p*s ≥ 0.054). Furthermore, the 2 (group: PLC/PLC2) * 2 (task: pro-/anti-saccade) * 6 (stimuli: angry/sad/fearful/happy/neutral/shapes) mixed ANOVA on latency was conducted. No group related main effect or interaction effects were observed (all *p*s ≥ 0.09).

**General effects of task and condition on performance**

For error rates, the treatment (OXT/PLC) * condition (social/non-social) * task (pro-/anti-saccade) mixed ANOVA found a significant main effect of condition (F_1, 68_ = 17.54, *p* < 0.001, η^2^p = 0.21) and task (F_1, 68_ =115.45, *p* < 0.001, η^2^p = 0.63), reflecting higher error rates for social compared to non-social stimuli (social: Mean ± SEM = 8.24% ± 0.68, non-social: Mean ± SEM = 6.00% ± 0.70), and higher error rates during the anti-saccade compared to the pro-saccade condition (anti: Mean ± SEM = 12.47% ± 1.10, pro: Mean ± SEM = 1.77% ± 0.29). Additionally, a significant interaction between condition and task was observed (F_1, 68_ = 13.01, *p* < 0.001, η^2^p = 0.16). Post-hoc Bonferroni-corrected comparisons found increased error rates for social compared to non-social stimuli during anti-saccade rather than pro-saccade condition (anti: social: Mean ± SEM = 14.77% ± 1.27, non-social: Mean ± SEM = 10.16% ± 1.21, *p* < 0.001; pro: social: Mean ± SEM = 1.71% ± 0.23, non-social: Mean ± SEM = 1.84% ± 0.43, *p* = 0.73). This is in line with our previous findings (Xu et al., 2019) and suggests social stimuli and task modulate error rates.

For response latencies, the treatment * condition * task mixed ANOVA showed a significant main effect of condition (F_1, 68_ = 124.21, p < 0.001, η^2^p = 0.65) and task (F_1, 68_ = 979.00, *p* < 0.001, η^2^p = 0.94), with faster latencies for social compared to non-social stimuli (social: Mean ± SEM = 223.72 ± 2.20 ms, non-social: Mean ± SEM = 238.22 ± 2.51 ms) and increased anti-saccade compared to pro-saccade latencies (anti: Mean ± SEM = 272.36 ± 2.92 ms, pro: Mean ± SEM = 189.58 ± 2.29 ms). Additionally, a significant interaction between condition and task was observed (F_1, 68_ = 128.08, *p* < 0.001, η^2^p = 0.65), with post-hoc Bonferroni-corrected comparisons showing faster latencies for social compared to non-social stimuli during the pro-saccade (social: Mean ± SEM = 175.58 ± 2.20 ms, non-social: Mean ± SEM = 203.58 ± 2.68 ms, *p* < 0.001) but not the anti-saccade condition (social: Mean ± SEM = 271.86 ± 2.96 ms, non-social: Mean ± SEM = 272.86 ± 3.15 ms, *p* = 0.58).

**Measurements for potential confounders**

To control for potential confounders between different treatment groups subjects were asked to complete validated questionnaires in Chinese before drug treatment including scales measuring mood (PANAS), state and trait anxiety (STAI), depression (Beck Depression Inventory - BDI, Beck et al., 1996), autistic traits (Autism Spectrum Quotient - ASQ, Baron-Cohen et al., 2001), social anxiety (Liebowitz Social Anxiety Scale - LSAS, Heimberg et al.,1999 and Social Interaction Anxiety Scale - SIAS, Mattick and Clarke, 1998), childhood experience (Childhood Trauma Questionnaires - CTQ, Bernstein et al., 1998) and cognition and emotion regulation (Behavioral Inhibition System and Behavioral Activation System Scale - BIS/BAS, Carver and White, 1994; Action Control Scale - ACS, Kuhl, 1994; Cognitive Emotion Regulation Questionnaires - CERQ, Garnefski et al., 2001).

**Differences between oral and intranasal OXT group in mood and personality trait scores**

Independent t-tests were used to examine the potential confounding effects of mood and personality and no significant differences were found between oral and intranasal OXT group (all *ps* ≥ 0.06). The demographics and questionnaire scores for subjects in both groups were presented in **Table S1**.

**Non-significant hypothesis test for oral and intranasal OXT’s effect on top-down attentional control and anxiety**

To further examine the non-significant hypothesis that oral and intranasal OXT have similar effects on top-down attentional control, Bayesian analysis was conducted using JASP. A Bayesian independent-sample t-test was performed on the anti-saccade error rates in the oral and intranasal OXT groups. A Bayes factor (BF_01_ = 3.62) was observed, suggesting that the null hypothesis for the differences between oral and intranasal OXT was moderately fitted relative to the alternative model (Jeffreys, 1998). A similar Bayesian analysis on saccade latencies only revealed an anecdotal fit however (BF_01_ = 2.78), suggesting that there might be some differences between the effects of oral and intranasal OXT.

In addition, to explore whether oral and intranasal OXT have similar anxiolytic effects, the non-significant hypothesis was examined using a Bayesian independent-sample t test on the changes of SAI scores (post- minus pre-task SAI scores) via the oral and intranasal route. Results showed a Bayes factor (BF_01_ = 3.97), indicating that the non-significant differences between oral and intranasal OXT group were moderately fitted in the present data (Jeffreys, 1998).

**Control analysis**

To examine oral OXT’s effect on error rates data after the BOX-COX transformation, the treatment (oral OXT/PLC) * condition (social/non-social) * task (pro-/anti-saccade) mixed ANOVA was conducted. The results showed a significant main effect of treatment (F_1, 68_ = 4.06, *p* = 0.048, η^2^p = 0.06), represented as higher error rates after oral OXT compared to PLC treatment (PLC: Mean ± SEM = 1.83 ± 0.15, OXT: Mean ± SEM = 2.25 ± 0.15, Cohen’s d = 0.48). We also found a significant treatment * task interaction effect (F_1, 68_ = 5.21, *p* = 0.026, η^2^p = 0.07) with post-hoc Bonferroni-corrected comparisons showing that compared to PLC treatment, OXT increased error rates in the anti-saccade but not pro-saccade task (anti: PLC: Mean ± SEM = 3.06 ± 0.24, OXT: Mean ± SEM = 3.83 ± 0.24, *p* = 0.028, Cohen’s d = 0.54; pro: PLC: Mean ± SEM = 0.61 ± 0.09, OXT: Mean ± SEM = 0.67 ± 0.09, *p* = 0.63). Neither treatment * condition interaction effect (F_1, 68_ = 0.05, *p* = 0.82) nor treatment * condition * task interaction were significant (F_1, 68_ = 0.04, *p* = 0.84). An additional mixed ANOVA including treatment, task and stimuli (angry/sad/fearful/ happy/neutral faces and shape) on error rates to explore any stimulus-specific effects also did not find a significant treatment * task * stimuli interaction (F_5, 340_ = 0.34, *p* = 0.85) indicating that OXT did not produce different effects across individual stimuli.

To compare oral and intranasal OXT’s effect on anti-saccade error rates after the BOX-COX transformation, the treatment (intranasal OXT/oral OXT/intranasal PLC/oral PLC) * condition (social/non-social) mixed ANOVA we performed. Consistently, the results also showed a main effect of treatment (F_3, 132_ = 5.04, *p* = 0.002, η^2^p = 0.10) and significant differences between oral PLC and intranasal OXT (*p* = 0.006), intranasal PLC and OXT (p = 0.021) but not between oral and intranasal OXT (*p* > 0.99) were found in the post-hoc Bonferroni-corrected tests. In addition, the interaction effect between treatment and condition was also not significant (F_3, 132_ = 0.26, *p* = 0.86). In addition, the mixed ANOVA including treatment and stimuli (angry/sad/fearful/happy/neutral faces and shapes) on error rates did not reveal a significant treatment * stimuli interaction (F_15, 660_ = 1.52, *p* = 0.11). This indicates that OXT administered via the different routes did not produce any significant differences in anti-saccade errors for any specific stimuli.

**Table S1**. Demographics and questionnaire scores for subjects in oral and intranasal OXT group included in the comparison analysis

|  | Intranasal OXT | Oral OXT | t-value | *p*-value |
| --- | --- | --- | --- | --- |
| Gender | 33 males | 35 males |  |  |
| Age (Mean ± SEM) | 21.85 ± 0.45 | 22.46 ± 0.30 | 1.14 | 0.26 |
| **Pre-task** |  |  |  |  |
| [Positive and Negative Affect Schedule](https://www.sciencedirect.com/topics/medicine-and-dentistry/positive-and-negative-affect-schedule) (PANAS) |  |  |  |  |
| Positive | 27.76 ± 1.01 | 28.26 ± 1.12 | 0.33 | 0.74 |
| Negative | 16.00 ± 0.88 | 14.89 ± 0.87 | 0.90 | 0.37 |
| State-Trait Anxiety Inventory (STAI) |  |  |  |  |
| SAI | 37.45 ± 1.60 | 40.00 ± 2.00 | 0.99 | 0.33 |
| TAI | 39.58 ±1.34 | 41.97 ± 1.58 | 1.15 | 0.26 |
| Liebowitz Social Anxiety Scale (LSAS) |  |  |  |  |
| Avoid | 20.45 ± 2.04 | 21.63 ± 2.22 | 0.39 | 0.70 |
| Fear | 22.39 ± 2.34 | 23.94 ± 2.23 | 0.48 | 0.63 |
| Beck Depression Inventory (BDI-II) | 6.27 ± 1.08 | 9.23 ± 1.64 | 1.49 | 0.14 |
| Social Interaction Anxiety Scale (SIAS) | 49.58 ± 2.32 | 50.60 ± 2.58 | 0.29 | 0.77 |
| Autism Spectrum Quotient (ASQ) | 21.70 ± 0.97 | 20.46 ± 1.04 | 0.87 | 0.39 |
| Childhood Trauma Questionnaires (CTQ) | 39.91 ± 1.20 | 41.06 ± 1.45 | 0.61 | 0.55 |
| Behavioral Inhibition System and Behavioral Activation System Scale (BIS/BAS) |  |  |  |  |
| BAS – Reward Responsiveness | 6.91± 0.29 | 6.51 ± 0.28 | 0.98 | 0.33 |
| BAS - Drive | 7.94 ± 0.34 | 7.46 ± 0.31 | 1.05 | 0.30 |
| BAS – Fun Seeking | 10.09 ± 0.39 | 9.66 ± 0.38 | 0.80 | 0.43 |
| BIS – Behavioral Inhibition | 15.88 ± 0.56 | 15.83 ± 0.47 | 0.07 | 0.94 |
| Action Control Scale (ACS) |  |  |  |  |
| Failure | 6.00 ± 0.59 | 5.77 ± 0.48 | 0.30 | 0.76 |
| Decision | 6.88 ± 0.48 | 6.03 ± 0.49 | 1.22 | 0.23 |
| Performance | 8.69 ± 0.33 | 8.71 ± 0.39 | 0.05 | 0.96 |
| Cognitive Emotion Regulation Questionnaires (CERQ) | 46.69 ± 1.32 | 44.71 ± 1.29 | 1.07 | 0.29 |
| **Post-task** |  |  |  |  |
| [Positive and Negative Affect Schedule](https://www.sciencedirect.com/topics/medicine-and-dentistry/positive-and-negative-affect-schedule) (PANAS) |  |  |  |  |
| Positive | 22.33 ± 1.40 | 25.86 ± 1.23 | 1.89 | 0.06 |
| Negative | 12.12 ± 0.59 | 12.94 ± 0.75 | 0.85 | 0.40 |
| State Anxiety Inventory (SAI) | 34.24 ± 1.32 | 36.46 ±1.73 | 1.01 | 0.32 |

**Reference**

Baron-Cohen S, Wheelwright S, Skinner R, Martin J, Clubley E (2001) The autism-spectrum quotient (AQ): Evidence from asperger syndrome/high-functioning autism, malesand females, scientists and mathematicians. Journal of autism and developmental disorders 31:5-17.

Beck AT, Steer RA, Ball R, Ranieri WF (1996) Comparison of Beck Depression Inventories-IA and-II in psychiatric outpatients. Journal of personality assessment 67:588-597.

Bernstein DP, Fink L, Handelsman L, Foote J (1998) Childhood trauma questionnaire. Assessment of family violence: A handbook for researchers and practitioners.

Carver CS, White TL (1994) Behavioral inhibition, behavioral activation, and affective responses to impending reward and punishment: the BIS/BAS scales. Journal of personality and social psychology 67:319.

Garnefski N, Kraaij V, Spinhoven P (2001) Negative life events, cognitive emotion regulation and emotional problems. Personality and Individual differences 30:1311-1327.

Heimberg RG, Horner K, Juster H, Safren S, Brown E, Schneier F, Liebowitz M (1999) Psychometric properties of the Liebowitz social anxiety scale. Psychological medicine 29:199-212.

Jeffreys H (1998) The theory of probability: OUP Oxford.

Kuhl J (1994) Action versus state orientation: Psychometric properties of the Action Control Scale (ACS-90). Volition and personality: Action versus state orientation 47.

Mattick RP, Clarke JC (1998) Development and validation of measures of social phobia scrutiny fear and social interaction anxiety. Behaviour research and therapy 36:455-470.

Xu X, Li J, Chen Z, Kendrick KM, Becker B (2019) Oxytocin reduces top-down control of attention by increasing bottom-up attention allocation to social but not non-social stimuli–a randomized controlled trial. Psychoneuroendocrinology 108:62-69.
